# CD152 (CTLA-4) Determines CD4 T Cell Migration *In Vitro* and *In Vivo*

**DOI:** 10.1371/journal.pone.0005702

**Published:** 2009-05-27

**Authors:** Karin Knieke, Holger Hoff, Frank Maszyna, Paula Kolar, Arnhild Schrage, Alf Hamann, Gudrun F. Debes, Monika C. Brunner-Weinzierl

**Affiliations:** 1 Experimentelle Pädiatrie, Universitätskinderklinik – Otto-von-Guericke Universität, Magdeburg, Germany; 2 Deutsches Rheuma-Forschungszentrum Berlin and Medizinische Klinik mit Schwerpunkt Rheumatologie und Klinischer Immunologie, CCM, Charité -Universitätsmedizin Berlin, Berlin, Germany; 3 Department of Pathobiology, University of Pennsylvania, Philadelphia, Pennsylvania, United States of America; Beijing Institute of Infectious Diseases, China

## Abstract

**Background:**

Migration of antigen-experienced T cells to secondary lymphoid organs and the site of antigenic-challenge is a mandatory prerequisite for the precise functioning of adaptive immune responses. The surface molecule CD152 (CTLA-4) is mostly considered as a negative regulator of T cell activation during immune responses. It is currently unknown whether CD152 can also influence chemokine-driven T cell migration.

**Methodology/Principal Findings:**

We analyzed the consequences of CD152 signaling on Th cell migration using chemotaxis assays *in vitro* and radioactive cell tracking *in vivo*. We show here that the genetic and serological inactivation of CD152 in Th1 cells reduced migration towards CCL4, CXCL12 and CCL19, but not CXCL9, in a G-protein dependent manner. In addition, retroviral transduction of CD152 cDNA into CD152 negative cells restored Th1 cell migration. Crosslinking of CD152 together with CD3 and CD28 stimulation on activated Th1 cells increased expression of the chemokine receptors CCR5 and CCR7, which in turn enhanced cell migration. Using sensitive liposome technology, we show that mature dendritic cells but not activated B cells were potent at inducing surface CD152 expression and the CD152-mediated migration-enhancing signals. Importantly, migration of CD152 positive Th1 lymphocytes in *in vivo* experiments increased more than 200% as compared to CD152 negative counterparts showing that indeed CD152 orchestrates specific migration of selected Th1 cells to sites of inflammation and antigenic challenge *in vivo*.

**Conclusions/Significance:**

We show here, that CD152 signaling does not just silence cells, but selects individual ones for migration. This novel activity of CD152 adds to the already significant role of CD152 in controlling peripheral immune responses by allowing T cells to localize correctly during infection. It also suggests that interference with CD152 signaling provides a tool for altering the cellular composition at sites of inflammation and antigenic challenge.

## Introduction

The positioning of differentiated effector/memory T cells within tissues is important for the adaptive immune responses to ensure protection against reinfection. Chemokines and their receptors are essential for homing of antigen-experienced T cells to lymphoid and non-lymphoid destinations [1], [2]. The signals responsible for the chemokine-directed migration of certain T cell subsets remain poorly characterized.

Following initial activation, naïve CD4^+^ T cells can differentiate into Th1 cells that secrete pro-inflammatory cytokines such as IFN-γ and TNFα, and induce cell-mediated immune responses [3]. Th1 responses are critical for the eradication of intracellular pathogens such as *Leishmania major* and *Listeria monocytogenes*. However, Th1 responses also drive chronic inflammation and tissue destruction in autoimmune diseases such as multiple sclerosis, type I diabetes, Crohn's disease, and rheumatoid arthritis [4]–[8]. Given the requirement for trafficking of effector T cells to diverse tissue sites in the body, it seems likely that there is substantial heterogeneity in the migratory capacity of Th cell subsets.

CD28 and CD152 are the primary co-receptors on T cells that mediate positive and negative costimulation using the same ligands on APCs, CD80 and CD86. Triggering of CD28 strongly up-regulates IL-2 production and T cell proliferation, which is counter-regulated by CD152-mediated inhibition of IL-2 transcription and cell cycle progression [9]. CD152 does not only oppose CD28-mediated effects, but can also synergize with it, *e.g.* by enhancing resistance to apoptosis [10]–[13]. Thus, the role of CD152 is more complex than its traditional view as a major down-regulator of immune responses [9], [14], [15]. CD152 is inducible after stimulation of T cells and reaches maximal surface levels 48 h after T cell stimulation [16], when maximal CD152 function is observed. The regulation of CD152 surface expression represents a major control point for its function because only cell surface expressed CD152 is functional [11], [16], and only a restricted number of activated T cells can express CD152. CD152 is transported from intracellular vesicles to the cell membrane where it is expressed in a polarized manner at the immunological synapse [17]. The surface expression is mainly induced by TCR signaling strength, but can be enhanced by soluble factors [18], [19]. Costimulation is an important control point to ensure that professional APCs define initiation, differentiation, and persistence of T cell responses. It has recently been proposed that tissue specific APCs direct and reprogram T cell migration into specific tissues [20], [21]. Given the central role of costimulation in differentiation and function of T cells, we postulate that costimulatory molecules participate in the localization of adaptive immune responses by licensing T lymphocytes for chemotaxis. In the present study, we investigated the functional role of CD152 in migration of Th1 cells. We show that CD152 surface expression is induced in Th1 cells stimulated primarily by mature DCs when compared with other APCs. Surprisingly, CD152 engagement induced activated Th1 cell migration towards CXCL12, CCL19, and CCL4, the ligands for CXCR4, CCR7, and CCR5, respectively, whereas inhibition of CD152 abrogated migration. Importantly, CXCR4 and CCR5 and their ligands are associated with inflammatory diseases [22], [23] and CCL19 as well as CXCL12 are also important for lymphocyte homing to lymph nodes and Peyer's patches [24]–[26]. Moreover, we show that CD152-mediated signals endow effector T cells with the capacity to migrate to sites of inflammation and lymph nodes. This novel function of CD152 could explain the retention and directed migration of effector cells to secondary lymphoid organs and sites of antigenic-challenge and might be important for T cell homeostasis as well as Th1-dominated diseases.

## Results

### Serological and genetic inactivation of CD152 reduces chemotaxis of Th1 cells to CXCL12 and CCL4

**Figure 1:**
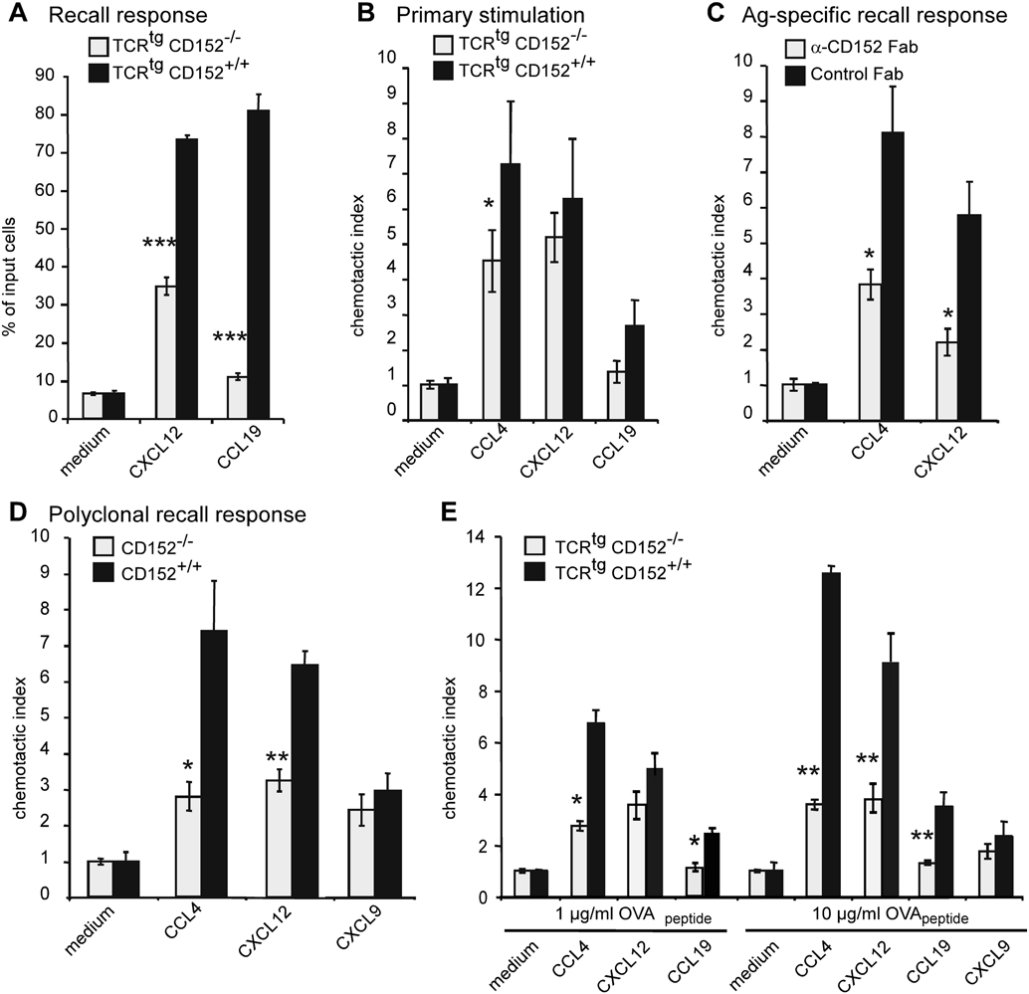
CD152 enhances chemotaxis of Th1 cells. (A) Migration of unpolarized T cells. Recall response of CD4^+^ OVA-specific TCR^tg^ T cells from CD152^−/−^ or CD152^+/+^ mice was induced by adding 1 µg/ml OVA_323–339_ and T cell-depleted APCs. On day 6 of recall response cells were analyzed in chemotaxis assays. Bars indicate migrated CD4^+^ cells as percentage of input cells. (B) Migration of CD152^−/−^ and CD152^+/+^ T cells in primary stimulation: CD4^+^ OVA-specific TCR^tg^ T cells were stimulated with 1 µg/ml OVA_323–339_ and T cell-depleted APCs. On day 6 of primary stimulation cells were analyzed in chemotaxis assays. Bars show the chemotactic index of CD4^+^ cells. (C) Specific migration of antigen-specific stimulated Th1 cells in a recall response: Primary stimulation and recall response of CD4^+^CD62L^+^ OVA-specific TCR^tg^ T cells were performed under Th1 conditions with 1 µg/ml OVA-peptide in the presence of 200 µg/ml neutralizing anti-CD152 Fab fragments or hamster control Fab fragments. Cells were examined in chemotaxis assay on day 6 of recall response. (D) Chemotactic index of a polyclonally induced recall response of CD152^−/−^ or CD152^+/+^ Th1 cells: Primary stimulation and recall response of splenocytes from CD152^−/−^ and CD152^+/+^ mice were induced polyclonally (as described in Material and Methods) and CD4^+^ cells were used for chemotaxis assays on day 4 of recall response. (E) Migration of CD152^−/−^ and CD152^+/+^ Th1 cells in a recall response is dose dependent: Primary stimulation and recall response of CD4^+^ cells from TCR^tg^ CD152^−/−^ and CD152^+/+^ mice were induced antigen-specifically using Th1 conditions with indicated amounts of OVA-peptide and cells were analyzed on day 6 of recall response in chemotaxis assays. All data shown represent one out of 3–4 similar experiments.

To examine the role of CD152 in T cell migration, we stimulated CD152-deficient (CD152^−/−^) monoclonal OVA-specific TCR^tg^ CD4^+^ T cells from OTII mice (TCR^tg^CD152^−/−^) and monoclonal OVA-specific TCR^tg^ CD4^+^ T cells from CD152^+/+^ OTII mice (TCR^tg^CD152^+/+^) with cognate antigen *in vitro* and tested chemotaxis to the CXCR4 ligand CXCL12, the CCR7 ligand CCL19, and the CCR5 ligand CCL4 in a Transwell chemotaxis assay (Fig. 1A, and data not shown). Similar baseline migration, which was routinely 5–10% in our Transwell migration assays (Figs. 1–5), was detectable for TCR^tg^CD152^+/+^ T cells and TCR^tg^CD152^−/−^ T cells. Under these conditions, no migration towards the CCR5 ligand CCL4 was seen in either population (data not shown). In contrast, TCR^tg^CD152^+/+^ T cells migrated 2-fold better in response to CXCL12 than did TCR^tg^CD152^−/−^ T cells (Fig. 1A). Similarly, migration towards the CCR7 ligand CCL19 was diminished by 70% in TCR^tg^CD152^−/−^ T cells compared with TCR^tg^CD152^+/+^ cells.

**Figure 2 pone-0005702-g002:**
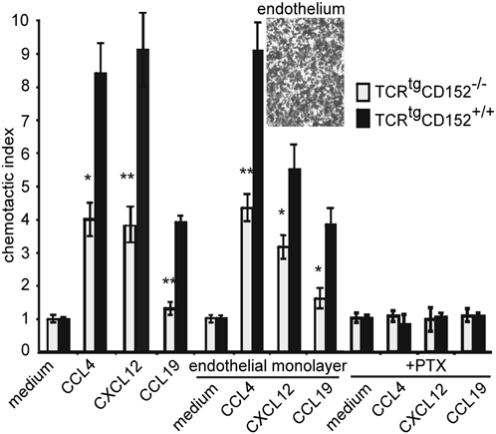
Only CD152^+/+^ but not CD152^−/−^ cells show efficient transendothelial migration. Primary stimulation and recall response of CD4^+^ OVA-specific TCR^tg^ T cells from CD152^−/−^ and CD152^+/+^ mice were performed using Th1 conditions with 10 µg/ml OVA-peptide and T cell-depleted APCs. On day 6 of recall response migration of CD4^+^ cells through membranes coated with or without endothelial monolayer was analyzed after 90 min. incubation at 37°C in Transwell chemotaxis assay. CD152 mediated migration of Th1 cells is G-Protein dependent: CD4^+^ OVA-specific TCR^tg^ CD152^−/−^ and CD152^+/+^ Th1 cells were incubated for 2 hours at 37°C in the presence of 100 ng/ml Pertussis toxin prior to examination in chemotaxis assay. (Inset) Endothelial cells (mlEND) were grown to a confluent monolayer for 48 h on Transwell membranes and confluency was controlled by microscopy. Shown data represent one out of 2 similar experiments.

**Figure 3 pone-0005702-g003:**
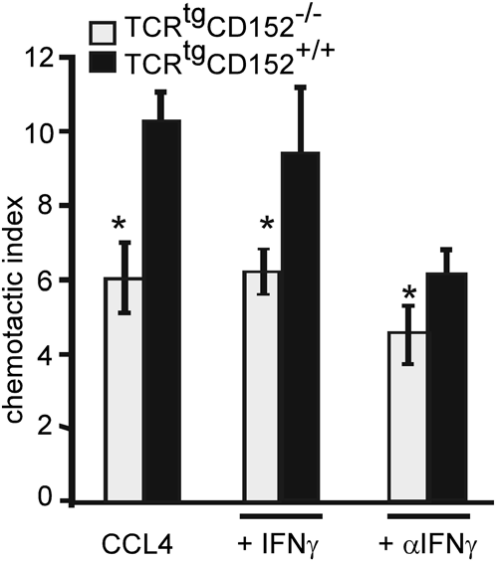
CD152-enhanced migration is IFNγ independent. CD4^+^ OVA-specific TCR^tg^ splenocytes from CD152^−/−^ and CD152^+/+^ were stimulated under Th1 conditions (as described in Fig. 2) in the presence of 20 ng/ml recombinant IFN-γ or 10 µg/ml blocking anti-IFN-γ -antibodies. On day 6 after inducing a recall response cells were analyzed for migration towards CCL4 in chemotaxis assays. The data represent one out of 3 similar experiments.

**Figure 4 pone-0005702-g004:**
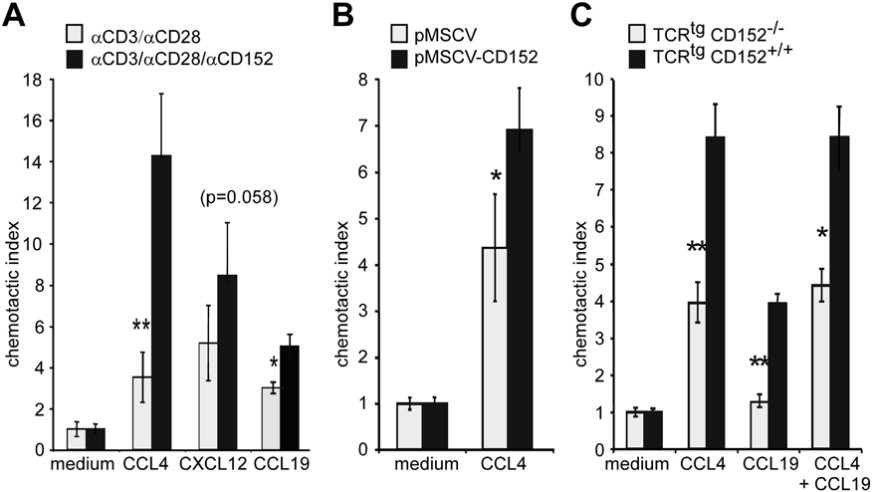
CD152-signaling mediates migration of recently activated Th1 cells. (A) CD4^+^CD62L^+^ TCR^tg^ T cells were stimulated with 1 µg/ml OVA peptide and T cell-depleted APCs under Th1 conditions. Recall response of Th1 cells was induced by platebound anti-CD3/anti-CD28 for 48 hours. Cells were washed, incubated with microspheres coated with anti-CD3, anti-CD28 and anti-CD152 (0.15 µg/ml, 0.4 µg/ml, 4.5 µg/ml respectively) or anti-CD3, anti-CD28 and hamIgG for additional 24 hours and examined in chemotaxis assay. (B) Restored specific migration of CD152^−/−^ Th1 cells by CD152 expression. CD152^−/−^ splenocytes were activated with 2 µg/ml anti-CD3 under Th1 conditions and on day 2 were retrovirally transduced with cDNA of full length CD152 (pMSCV-CD152) or with empty vector pMSCV. Chemotactic capacity of transduced CD4^+^ cells was determined in chemotaxis assays. (C) Migration towards CCL19 plus CCL4. CD4^+^ TCR^tg^ T cells from CD152^+/+^ and CD152^−/−^ mice were stimulated under Th1 conditions as described in figure 2. On day 6 of recall response viable cells were analyzed in chemotaxis assays for migration capacity towards CCL4 and CCL19 alone or in combination. One out of at least 3 experiments with similar results is shown.

**Figure 5:**
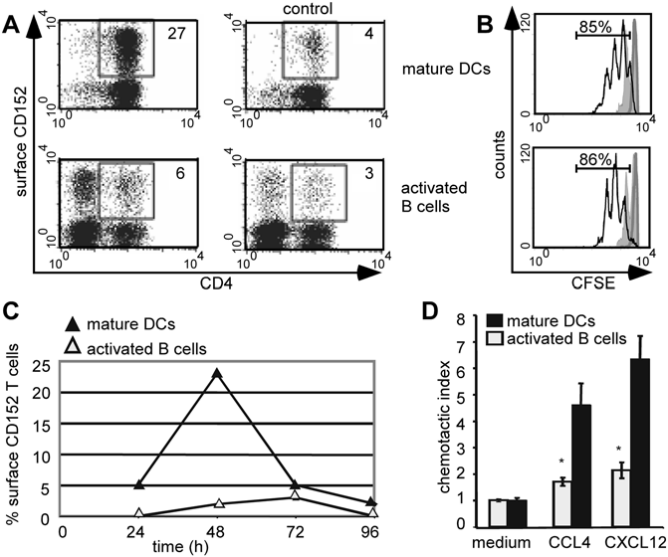
CD152-enhanced migration is primarily mediated by DCs. (A) Surface expression of CD152 on CD4^+^ cells is up-regulated by stimulation with DCs. Naϊve CD4^+^CD62L^+^ TCR^tg^ T cells were stimulated with 1 µg/ml OVA-peptide presented by matured DCs or by LPS-activated B cells. After 48 hours surface expression of CD152 was detected by liposome staining technique and subsequent FACS analysis. Left panels show CD152 staining, right panels show blocking controls. Numbers indicate the percentage of CD152^+^ CD4^+^ based on total CD4^+^ cells. (B) Equal proliferation of T cells stimulated with activated B cells or matured DCs. Naϊve CD4^+^CD62L^+^ TCR^tg^ T cells were labeled with CFSE and antigen-specifically stimulated with activated B cells or matured DCs. Proliferation of T cells was determined by flow cytometry 48 hours (filled curve) and 72 hours later (black line). T cells cultured with different APCs but without antigenic stimulation are shown as heavy grey line. Numbers indicate the percentage of divided T cells 72 hours after stimulation relating to unstimulated controls (after 48 h 48% of Dc-stimulated T cells and 46% of Bc-stimulated T cells proliferated). (C) Kinetics of CD152 surface expression on CD4^+^ T cells. Indicated time after onset of stimulation cells cultured as described in 5A were stained with liposome staining technique for CD152. Percentages of surface expressing CD152 cells of total CD4^+^ cells are shown. (D) Chemotactic index of Th1 cells stimulated with different APCs. CD4^+^CD62L^+^ TCR^tg^ T cells were stimulated under Th1 conditions with 1 µg/ml OVA peptide presented by activated B-cells or bone marrow derived DCs. On day 6 of primary stimulation, CD4^+^ cells were analyzed for migration capacity in chemotaxis assay. All data show representative data from at least two experiments.

To examine a role for CD152 in the contribution to the migratory properties of pro-inflammatory T lymphocytes upon primary stimulation, CD152^−/−^ and CD152^+/+^ TCR^tg^ CD4^+^CD62L^+^ T lymphocytes were stimulated with antigen under Th1 conditions and migration was evaluated in Transwell chemotaxis assays on day 5 (data not shown) or day 6 after stimulation (Fig. 1B); both assays gave similar results. Cells capable of receiving a CD152 signal showed a significantly higher migratory response toward the inflammatory chemokine CCL4 (Fig. 1B). In contrast, for the mainly homeostatic chemokines CXCL12 and CCL19, no significant difference in migration was observed when comparing monoclonal CD4 cells from TCR^tg^CD152^+/+^ and TCR^tg^CD152^−/−^ mice.

CD152 functions are more pronounced upon activation of antigen-experienced T cells than upon primary stimulation of naïve T cells [27]. Therefore, we inactivated CD152 on antigen-experienced Th1 cells by adding neutralizing anti-CD152 Fab fragments to Th1 cells during primary stimulation and recall response, and monitored migration on day 6 of recall response (Fig. 1C). Polarization of primary Th1 cells resulted in >98% pure effector populations, as confirmed by a CD44^high^ IFN-γ^+^ IL-4^−^ phenotype (data not shown). Following recall responses of antigen-experienced Th1 cells, cells that received a CD152 signal migrated two-fold better towards CCL4 than Th1 cells that were activated under a serological blockade of CD152 (Fig. 1C). Similarly, blockade of CD152 by the addition of anti-CD152 Fab fragments also resulted in reduced frequencies of cells migrating towards CXCL12. To substantiate a role for CD152 in mediating the migratory capacity of antigen-experienced T lymphocytes, we used T cells from CD152^−/−^ and CD152^+/+^ mice. On day 4 after a polyclonally induced recall response of splenocytes with anti-CD3 and APCs, substantially more CD152^+/+^ T cells migrated compared with CD152^−/−^ T cells (Fig. 1D): wild-type cells migrated three- and two-times better than CD152^−/−^cells toward CCL4 and CXCL12, respectively. Intriguingly, using 10 µg/ml instead of 1 µg/ml OVA during stimulation enhanced the migration for TCR^tg^ CD152^+/+^ T cells by 2-fold (Fig. 1E). In contrast, CD152^−/−^ T cells did not migrate significantly better with higher peptide concentrations (Fig. 1E). Thus, the enhanced migration of CD152^+/+^ T cells towards CCL4 and CXCL12 was dependent on the concentration of antigen during TCR stimulation (duration and strength of signal). Interestingly, this chemotaxis enhancing property was specific to CCL4 and CXCL12, because no difference in migration towards CXCL9 occurred, even when high OVA concentrations were used for stimulation. Migration towards CCL19 was slightly enhanced by altering the signaling strength during stimulation, when Th1 cells received a CD152 signal (Fig. 1E). Most strikingly, CD152^+/+^ but not CD152^−/−^ antigen-experienced Th1 cells migrated to the homeostatic chemokine CCL19.

In summary, the presence of functional CD152 enhanced the chemotaxis of T cells, especially that of repeatedly activated Th1 cells.

### CD152 enhances chemokine-driven transendothelial migration

Recruitment of lymphocytes to the site of infection involves a sequential, multi-step process leading to the active *transmigration* of lymphocytes across the blood vasculature endothelium [28]. As part of the process, lymphocytes migrate through endothelial cell junctions, a step referred to as diapedesis. Using mlEND endothelial cells grown confluently on a membrane (Fig. 2 inset), transmigration of Th1 cells towards CCL4, CXCL12 and CCL19 across an endothelial layer was compared to transmigration across a membrane only (Fig. 2). Migration of CD152^+/+^ Th1 cells towards indicated chemokine gradients of CCL4, CXCL12 and CCL19 was enhanced at least two-fold as compared with CD152^−/−^ Th1 cells, no matter whether transmigration was performed across a membrane only or across an endothelial layer. Thus, we conclude that CD152 engagement enhances transendothelial migration of T cells (Fig. 2).

### CD152-induced migration of pre-activated Th1 cells depends on G_αi_ signaling and is independent of IFN-γ

Chemokine receptors are known to signal through G proteins [29]. By blocking G***_α_***_i_-linked chemokine receptor signaling with pertussis toxin (PTX), CD152-stimulated migration was reduced to that of background migration (Fig. 2). Thus, CD152-enhanced migration of T cells completely depends on G***_α_***_i_ signal transduction.

To control for the possibility that increased IFN-γ levels of CD152^−/−^ T cell populations contributed to the reduced migration [11], [30], the IFN-γ-production was examined during *in vitro* culture. Similar IFN-γ-production was detected in TCR^tg^CD152^−/−^ and TCR^tg^CD152^+/+^ CD4^+^ T cells under Th1 conditions and with strong antigenic stimulus (10 µg/ml OVA-peptide; data not shown). Furthermore we inhibited IFN-γ by adding neutralizing anti-IFN-γ to the cultures during the antigenic recall response. Adding anti-IFN-γ led to an overall reduction in migration, however, the reduced migration of TCR^tg^CD152^−/−^ Th1 cells could not be reversed by blocking IFN-γ (Fig. 3). Furthermore, addition of exogenous IFN-γ to the cultures did not reduce migration of CD152^+/+^ Th1 cells, indicating that CD152-modulated migration of Th1 cells was not due to effects of different IFN-γ concentrations between cultures of TCR^tg^CD152^−/−^ Th1 cells and TCR^tg^CD152^+/+^ Th1 cells.

### CD152 engagement induces chemokine responsiveness of recently activated T lymphocytes

CD152 exerts its main function during its peak expression in a T cell response, which is typically 48 h following activation [11], [16]. To monitor CD152-mediated effects on T cell migration, a recall response of Th1 cells was induced for 48 h with congenic APCs and OVA. Additionally, antibody-coupled microspheres were used to cross-link CD152 in combination with CD3 and CD28 coligation. The stimulation with anti-CD3/anti-CD28-coupled microspheres allowed for direct crosslinking of CD152 and also kept the CD28-signal constant in all populations. On day 3 of activation, migration of activated CD152-engaged Th1 cells towards CXCL12 and CCL19 was considerably enhanced compared with Th1 cells engaged only with CD3 and CD28 (Fig. 4A). Strikingly, 4-times more Th1 cells migrated towards CCL4 after stimulation with anti-CD3/anti-CD28/anti-CD152 compared with anti-CD3/anti-CD28-stimulated T cells. Thus, CD152 engagement in concordance with CD3 and CD28 of Th1 cells triggered chemokine responsiveness in the absence of additional signals provided by APCs.

To confirm that enhanced migration of Th1 cells is regulated by CD152 and is not merely the result of an early differentiation event, we retrovirally transduced CD152^−/−^ cells during *in vitro* stimulation with the cDNA of full length CD152 (pMSCV-CD152). Upon a recall response, retroviral transduction with pMSCV-CD152, but not with the control vector, restored migration towards CCL4 in CD152^−/−^ cells (Fig. 4B). Thus, CD152 expression mediates enhanced migration even in highly activated T cells and the detected lack of migration of CD152^−/−^ T cells is due to their lack of CD152 and not to developmental alterations.

Next, we determined whether the CD152-controlled migration towards the inflammatory chemokine CCL4 and the homoeostatic chemokine CCL19 reflects heterogeneity of the T cell population. When migration towards a single chemokine gradient of CCL4 or CCL19 was tested, TCR^tg^CD152^+/+^ Th1 cells showed enhanced migration compared with TCR^tg^CD152^−/−^ Th1 cells after antigen-specific recall responses (Fig. 4C). Migration to a combined chemokine gradient of CCL4 and CCL19 was similar to migration towards a single CCL4 gradient, suggesting that the same cell population responds upon CD152-signaling towards CCL4 and CCL19.

### CD152 is predominantly expressed on the surface of T cells stimulated by DCs

CD152 is not expressed on the surface of resting T cells and is difficult to detect even after stimulation of T cells [18], [31], [32]. In order to analyze CD152 expression on individual T cells activated by different APCs we stimulated naïve OVA-specific CD4^+^ T lymphocytes with the peptide OVA_323–339_ loaded on either activated B lymphocytes or DCs. To unambiguously identify T cells expressing surface CD152, we used an enhanced staining technique [33] based on CD152-specific immunofluorescent liposomes, which increased the detection sensitivity at least 1000-fold [11], [16]. Employing this method, expression of surface CD152 was evaluated at various time points after T cell activation (Fig. 5A, C). B cells and DCs stimulated T cells equally well as determined by the induction of similar T cell proliferation rates of 85–86% (Fig. 5B) on day 3 after onset of stimulation. Specific surface expression of CD152 was detected with low frequencies on day 1 in primary T cell cultures activated with either activated B cells or DCs. Following primary stimulation, CD152 expression increased and peaked after 48 h. Thereafter, the percentage of surface CD152-expressing cells declined in both cultures and these cells were infrequent (<3%) after 3 days of stimulation. At the peak of surface CD152 expression, T cell populations stimulated by DCs displayed enhanced expression of surface CD152. Whereas more than 23% of the T cells stimulated by DCs expressed surface CD152, only ∼3% of the T cells displayed CD152 on their surface 48 h after stimulation with activated B cells (Fig. 5A, C). The number of surface CD152-expressing cells could not be attributed to contaminating Th2 cells, as no IL-4 producers were present in the cultures (data not shown). Thus, mainly T cells stimulated by DCs are able to express surface CD152, showing that the activation history and activating cell type (DC versus B cell) regulate CD152 expression on T cells.

Next, we asked whether the differential potential of DCs versus activated B cells to induce CD152 on T cells correlated with a differential potential to license T cells for chemotaxis. Correlating with the induction potential of CD152 on T cells, T cells stimulated by DCs migrated significantly better in response to CCL4 or CXCL12 than did T cells activated by B cells (Fig. 5D). Thus, the data suggest that chemokine-driven migration of T cells is controlled by mature DCs and correlates tightly with the up-regulation of surface CD152 on T cells.

### CD152 induces expression of chemokine receptors CCR5 and CCR7

**Figure 6:**
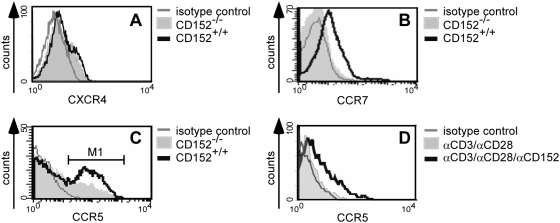
CD152 mediates up-regulation of chemokine receptors CCR5 and CCR7. Recall response of splenocytes from CD152^+/+^ and CD152^−/−^ mice was induced polyclonally under Th1 conditions. On day 3 of recall response, cells were stained for surface markers and analyzed by flow cytometry. The histograms indicate surface expression on CD4^+^ lymphocytes of (A) CXCR4, (B) CCR7 and (C) CCR5; (M1: 48% CD152^+/+^; 29% CD152^−/−^). (D) After performing recall response of splenocytes from CD152^+/+^ mice with microspheres (as described in Fig. 4A) CCR5 was detected on CD4^+^ cells. Representative results from one out of 3–5 experiments are shown.

Many reports show that CD152 down-regulates expression of effector molecules such as IL-2 and cyclin-dependent kinases [9], [34]. We have shown recently that CD152 can indeed up-regulate the expression and activate molecules such as Bcl-2, pFKHRL1, and PI3'K [11]. To determine the effects of CD152 on the expression of chemokine receptors, cells from CD152^−/−^ and CD152^+/+^ mice were used. After polyclonal stimulation of splenocytes from CD152^−/−^ and CD152^+/+^ mice with anti-CD3 and induction of recall response with syngeneic APCs and anti-CD3, we detected no difference in CXCR4 expression (Fig. 6A), but we found enhanced expression of CCR7 by CD152^+/+^ cells compared with CD152^−/−^ cells (Fig. 6B). To further elucidate the role of CD152 in the induction of CCR5, splenocytes of CD152^−/−^ and CD152^+/+^ mice were activated with anti-CD3 under Th1 polarizing conditions, and upon recall response, analyzed for CCR5 expression on CD4 cells. 20% more CD152^+/+^ than CD152^−/−^ CD4 T cells expressed CCR5 on their cell surface (Fig. 6C). In addition, agonistic signaling of CD152 in CD152^+/+^ T lymphocytes using anti-CD3/anti-CD28/anti-CD152 cross-linking under Th1 conditions up-regulated CCR5 as compared to T cell activation with only anti-CD3/anti-CD28 cross-linking (Fig. 6D). Thus, CD152 regulates expression of the chemokine receptors CCR5 and CCR7 correlating with the observed CD152-enhanced migration to the corresponding ligands CCL4 and CCL19.

### Differential migration pattern of CD152^+/+^ and CD152^−/−^ Th1 cells in vivo

**Figure 7:**
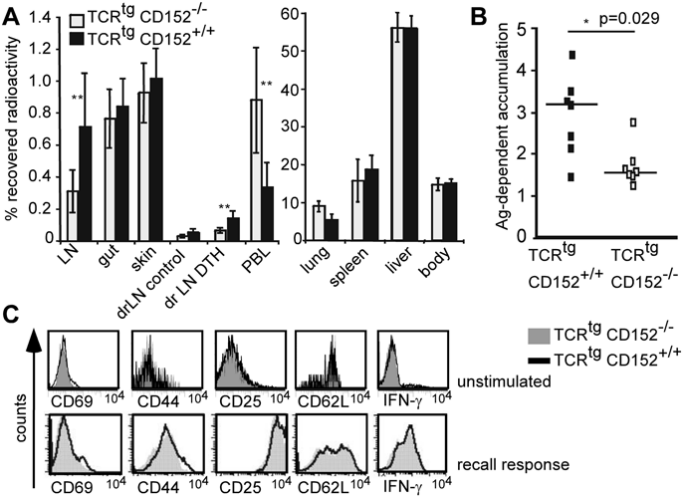
Th1 cell homing to the site of inflammation and lymph nodes is enhanced by CD152. (A) Distribution of TCR^tg^ CD152^−/−^ and CD152^+/+^ Th1 cells 48 hours after transfer. *In vitro* induced Th1 cells from a recall response were radioactively labeled and injected i.v. (5×10^6^ cells per mouse). After 24 hours OVA-peptide (250 ng) in IFA was administered s.c. into one footpad (DTH); PBS/IFA-injection in the other footpad served as control. Different organs were analyzed after 48 hours for radioactivity. Recovered radioactivity in organs was calculated as percentage of total amount of radioactivity per mouse (7 mice per group). (LN: mesenteric and axillary lymph nodes; drLN: draining (popliteal and inguinal) lymph nodes; PBL: peripheral blood lymphocytes). Results from one out of two similar experiments are shown. (B) Specific migration of transferred CD152^−/−^ and CD152^+/+^ Th1 cells to the site of inflammation. Radioactively labeled CD152^−/−^ and CD152^+/+^ Th1 cells were transferred into mice and DTH was induced in footpads as described in 7A. Shown is the antigen-dependent accumulation of Th1 cells to the site of inflammation indicating the ratio of recovered radioactivity in the inflamed footpad versus the PBS injected footpad. (C) Similar expression of activation induced markers and similar amount of IFN-γ-producers. TCR^tg^ CD152^−/−^ and CD152^+/+^ CD4^+^ T cells were either left unstimulated or were *in vitro* stimulated under Th1 conditions and a recall response was performed. Unstimulated cells were stained on day 3 (upper panel) and stimulated cells on day 5 of recall response for activation induced molecules (lower panel). Intracellular staining for IFN-γ of fixed cells was performed on day 3 of recall response after restimulation with PMA/Ionomycin for 6 hours; Brefeldin A was added for the last 2 hours of incubation. One out of two similar experiments is shown.

To test the *in vivo* significance of the enhanced *in vitro* migration towards inflammatory and lymphoid chemokines due to CD152 signaling, we analyzed the *in vivo* homing capacity of CD152^+/+^ and CD152^−/−^ TCR^tg^ Th1 cells. Recipient mice were either injected i.v. with radioactively labeled CD152^+/+^ TCR^tg^ Th1 or CD152^−/−^ TCR^tg^Th1 cells. 24 h after cell transfer, mice were injected s.c. into one footpad with OVA-peptide in IFA and in a control footpad with PBS in IFA only. 24 h later, radioactivity in different organs, the inflamed and accordingly control footpad was measured (Fig. 7A). Both transferred cell populations accumulated with no difference in non-inflamed organs (including gut, skin, lung, spleen, liver, rest of the body). Strikingly, CD152^+/+^ Th1 cells migrated enhanced into resting and inflammation draining lymph nodes compared with CD152^−/−^ Th1 cells (Fig. 7A). Importantly, CD152^+/+^ Th1 cells migrate 2-fold better into the site of inflammation (footpad) than did CD152^−/−^ Th1 cells (Fig. 7B). In independent experiments testing migration in the absence of inflammation, CD152^+/+^ Th1 cells also homed better than their CD152^−/−^ counterparts into lymph nodes (p| = |0.0006; data not shown). The detected differences in migration were not due to differences in polarization or activation status of CD152^−/−^ versus CD152^+/+^ T cells since both subsets expressed similar levels of the activation markers CD69, CD44, CD25, CD62L, had an identical capacity to secrete IFN-γ before stimulation (Fig. 7C, upper panel) and on day 5 after induction of recall response (Fig. 7C, lower panel) and showed no IL4-, IL10- or IL17-producing T cells at the time point of analysis in chemotaxis assays (data not shown). Consistent with their *in vitro* migration to inflammation (CCL4, CXCL12) and lymph node expressed chemokines (CCL19, CXCL12) CD152 competent T cells are licensed for migration into both, sites of acute inflammation and lymph nodes.

## Discussion

Our results show that CD152 expression and signaling in T cells is a major control point for their migration both *in vitro* and *in vivo*. The heterogeneity of CD152 expression in individual primary T cells stimulated by different APCs has not been analyzed previously and our study reveals that T cells stimulated by different APCs also differ in CD152-mediated enhanced *in vitro* chemotaxis. Mainly DC-stimulated effector T cells up-regulated surface CD152 and as a consequence migrated in response to CCL4 and CXCL12. Importantly, CD152 signals also enhanced Th1 cell migration into lymph nodes and a site of cutaneous inflammation in a short term *in vivo* homing assay. Thus, CD152 ligation determines the positioning of inflammatory T cells at the site of inflammation *in vivo*.

Expression of CD152 on the surface of primary T cells is difficult to detect using conventional cytometric and microscopic techniques [16], [18], [35], [36]. Consequently, most studies on CD152 expression have used intracellular staining methods. In contrast, the sensitive liposome-based staining technique used in this study allowed the detection of as few as 100 to 200 surface molecules per cell [37]. Using this method, we showed that the type of activating APC determines the frequency of surface CD152 expressing T cells. Interestingly, mature DCs, but not activated B cells, were potent in up-regulating surface CD152 on T cells. The fact that differentially activated (i.e. DC vs. activated B cell) T cells differentially express CD152 during an immune response raised the possibility that surface CD152^+^ and surface CD152^no/low^ T cells could also be heterogeneous in their migratory potentials. Indeed, many more DC-activated T cells expressed surface CD152 and were able to chemotax than were B-cell activated T cells (Fig. 5). Thus, different classes of APC induce distinct T cell migration patterns via the differential induction of CD152 on T cells.

CD152 and CD28 share common B7 ligands. However, B7 expression is not limiting on APCs during an optimal immune response, enabling saturated engagement of both CD28 and CD152 [14], making a simple competition of CD152 over CD28 unlikely and dispensable in our experiments. Additionally, there is accumulating evidence that CD152 directly modulates proximal TCR signaling [38]. It is therefore unlikely that enhanced CD28 signaling occurs during serological or genetic blockade of CD152. Therefore, we conclude that CD152-enhanced migration of Th cells is not simply a result of impaired CD28 signaling.

Most studies showed that CD152 signaling down-regulates expression of molecules and, ultimately, the function of T cells. In addition, CD152 signaling can up-regulate the expression and activation of molecules such as TGFβ, Bcl-2, pFKHRL1, LFA1, and PI3'K [11], [39], [40]. Functionally, this means that cells expressing CD152 on the cell surface and receiving a CD152 signal are resistant against activation-induced cell death [11]. Additionally, CD152 signals in activated T cells lead to reduced adhesion and increased motility that result in shortened T cell-APC interactions within lymph nodes [38], [41]. This finding was interpreted as a mechanism to down-regulate the immune response [42]. Thus, our data add an important new piece to the puzzle by showing that CD152 signals license T cells for chemotaxis and migration into lymph nodes and inflammatory sites. In conclusion, CD152-signaling does not simply silence T cells, but rather enables them to actively contribute to the immune response by enhancing effector T cell migration *in vivo*.

We have now demonstrated that CD152 signaling enhances the cell surface expression of CCR5 and CCR7 on T cells. It is likely that up-regulation of CCR5 and CCR7 supports the CD152-mediated enhanced migration towards of the cognate ligands CCL4 and CCL19, respectively. However, this may not be the only mechanism. For example, we were not able to detect CXCR4 up-regulation in response to CD152 signaling, even though we detected enhanced migration to its ligand CXCL12 (Fig. 1A–E). Chemotaxis is not always accompanied by enhanced chemokine receptor expression on leukocytes because factors that influence chemokine receptor signaling additionally control the migratory capacity in response to chemokines [43]–[46]. Thus, CD152 could regulate T cell chemotaxis alternatively or additionally to the induction of chemokine receptors on T cells by controlling downstream signaling events.

Our study is the first report demonstrating that CD152 ligation as well as genetic or serological ablation of CD152 signaling affects chemotaxis of primary T cells. At first view, our data seems to be inconsistent with the observation that CD152^−/−^ mice show dramatic lymphocyte infiltration of tissues [14], [28], [35], [47]. However, in these studies a massive expansion of activated lymphocytes is seen; and the cells might just overflow throughout the body in shortage of space. Therefore, it is likely that lymphocytic infiltration of tissues is not only a consequence of directed migration. Alternatively, migration of the expanded cell populations into tissues might be fairly random, but as CD152^−/−^ cells express CCR7 only at low frequencies (Fig. 6B), and CCR7 expression has been shown recently to be a prerequisite for T cells to exit extralymphoid tissues [48], [49], they could be retained in peripheral tissues.

In this study, we have shown that enhanced avidity of the TCR determines migration of CD152^+/+^ T cells along chemokine gradients. This suggests that cells receiving the most optimal signals during stimulation show enhanced CD152 expression and are licensed to migrate. These data are consistent with work of Allison and colleagues, showing that T cells receiving a strong signal up-regulate CD152 [15], [18]. However, our results demonstrate that CD152 expressing T cells are not silenced by CD152-signaling but, on the contrary, are directed for migration, thereby driving and preserving the immune response.

Taking into account that CD152 induces resistance against activation-induced cell death, together with our new results that CD152 enhances chemotaxis of already activated, effector-cytokine producing T cells, we hypothesize that CD152 surface expression and signaling marks the “fittest” T cells out of a heterogeneous population of stimulated T cells [50]. Depending on the environment, the CD152-marked cells would home to secondary lymphatic organs or to the site of inflammation, probably depending on the stimulation history and the quality of the chemotactic gradient [51]. These cells will be allowed, via CD152 signaling to drive the immune response to fight pathogens immediately and to drive progressive T cell differentiation of T_CM_ cells. Thus, CD152 might actively determine the fate of an effector T cell.

Importantly, consistent with our data showing that CD152 up-regulates expression and function of CCR7, a receptor crucial for entry of resting T cells and Th1 cells from the bloodstream into lymph nodes [24], [26], [52], we found that migration of T cells into lymph nodes was enhanced by CD152 (Fig. 7A). Moreover, we found that CD152 signals promoted accumulation of Th1 cells at a site of cutaneous inflammation (Fig. 7B), which was most likely the consequence of the CD152-enhanced responsiveness to CCL4 and CXCL12 (Fig. 1E, 2) as both ligands are widely associated with inflammation [22], [23]. However, CCR7 ligands can also be up-regulated in chronic inflammation [53], [54] and others have shown a role for CCR7 ligands in T cell migration into inflamed non-lymphoid tissues [55]. Consequently, it is possible that CD152-regulated CCR7 expression contributes to effector T cell migration into both lymphoid organs and inflamed tissues.

Our data suggest that upon activation, T cells will not migrate efficiently into lymph nodes and inflamed tissues unless they up-regulate surface CD152. The migratory properties of the cell population are important to mount an adaptive immune response in the periphery. Thus, CD152 might be one master switch to determine that lymphocytes are in the right time at the right place.

## Materials and Methods

### Mice

Transgenic (tg) mice for OVA-specific TCR^tg/tg^ on BALB/c background (Do11.10, gift from Dennis Y. Loh, Washington University School of Medicine, St. Louis, MO) or C57/B6 background (OTII), CD152 knock-out mice on C57/B6 background (gift from James P. Allison, University of California at Berkeley, CA). All mice were bred under specific pathogen-free conditions at the Bundesinstitut für Risikobewertung (Berlin) and were used at the age of 5 to 10 weeks except for non-transgenic CD152^−/−^ mice, which were used at the age of 3 weeks. Animal studies were reviewed and approved by Landesamt für Gesundheit und Soziales, Berlin (G0075/02).

### Antibodies, cytokines and reagents

The following antibodies were used: anti-CD152 (UC10-4F10-11), anti-CD25 (7D4), anti-CD69 (H1.2F3), anti-IL4 (11B11), anti-CCR5 (C34-3448), anti-CXCR4 (2B11/CXCR4), ratIgG_1_ (R3-34), hamIgG_2_ (KLH, HA4/8), ratIgG_2c_, ratIgG_2b_ (A95-1) (all from BD Biosciences), anti-CCR7 (4B12, eBioscience) ratIgG_2a_ (eBioscience) coupled to FITC, PE or Cy5-conjugates. Anti-CD28 (37.51), anti-CD3 (145-2C11), anti-IL4 (11B11), anti-CD4 (GK-1.5/4), anti-CD62L (MEL-14), anti-CD44 (IM7), anti-TCR^tg^ (KJ1-26.1), anti-IFN-γ (AN18.17.24), anti-CD11c (N418), anti-CD152 (UC10-4F10-11) and Syrian hamster control antibody (560-31.1B9; kindly provided by J.P. Allison) were purified from hybridoma supernatants. Anti-CD152 Fab fragments were prepared with the Immunopure Fab preparation kit (Pierce) and used at 200 µg/ml. Recombinant chemokines and IL-12 were from R&D Systems, pertussis toxin from Sigma; magnetic microbeads (anti-CD4, anti-CD19, anti-CD43, anti-CD62L, anti-CD90, anti-FITC multisort) were from Miltenyi-Biotech; and sulfate polystyrene latex microspheres of 5 µm±0.1 µm mean diameters from Interfacial Dynamics.

### Cell isolation and stimulation

Isolation of naïve CD62L^high^CD4^+^ T cells was performed using magnetic cell separation (MACS™) to a purity greater than 98%. Isolated TCR^tg^ cells were stimulated with 1 or 10 µg/ml OVA_323–339_ peptide (provided by Schneider-Mergener, Charité) and T cell-depleted splenocytes as APCs. On day 6 after onset of stimulation, recall response was performed by adding freshly isolated APCs and OVA-peptide to density-gradient-purified cells. CD152^−/−^ or CD152^+/+^ TCR^wt^ splenocytes were stimulated with 1.5 µg/ml ConA and recall response was induced on day 5 with 2 µg/ml anti-CD3 and CD90^−^ APCs from syngeneic C57/B6 mice. CD43^−^ B cells were activated with 20 µg/ml LPS for 24 hours. DCs were generated from bone marrow of syngeneic mice by culturing for 8 days with 10% GM-CSF and subsequent maturation for 24 hours with TNFα (10 ng/ml). For Th1 polarization recombinant IL-12 (10 ng/ml) and anti-IL-4 (6 µg/ml) were added to the cultures. Crosslinking of CD152 on CD4 cells was performed using latex microspheres coated with antibodies against CD3 (0.15 µg/ml), CD28 (0.4 µg/ml), CD152 or a hamster control antibody (N418, 4.5 µg/ml) as described [16], [32]. CD4 T cells (1×10^6^/ml) were stimulated at a ratio of one bead per cell with antibody-coupled microspheres. Cell cycle progression was measured by labeling of T cells with CFSE (5 µM in PBS, Molecular Probes) for 5 min at room temperature. The reaction was stopped by washing and resuspending the cells in RPMI-1640 medium.

### Flowcytometric analysis of CD152 surface expression

Surface expression of CD152 was detected using magnetofluorescent liposomes [16], [33]. T cells were incubated with 1 µg/ml hamster CD152 antibody of for 15 min at 4°C. Cells were washed and incubated with Cy5 dye-filled liposomes, conjugated to hamster Fab fragments, for 30 min at 4°C. Unbound liposomes were removed by washing. The specificity of CD152 staining was controlled by isotype control antibody conjugated with Cy5-filled liposomes as well as by incubation of cells with Cy5-filled liposomes only.

### Retroviral transduction

293T fibroblasts were transfected with calciumphosphate-precipitated pECO, pCGP, pMSCV-GFP (empty vector) or pMSCV-GFP-CD152 (containing full length CD152 cDNA) respectively. After 4 hours medium was exchanged and fibroblasts were cultured for additional 48 hours in DMEM containing 10% FCS, 2 mM glutamine and 50 µM 2-mercaptoethanol. CD152^−/−^ splenocytes, activated with 2 µg/ml anti-CD3 using Th1 conditions for 1–2 days, were resuspended in virus supernatant and retrovirally transduced by centrifugation for 90 minutes at 25°C. Cells were resuspended in conditioned medium, recall response was performed on day 4 and cells were analyzed in chemotaxis assays 3 days later.

### Chemotaxis assay

Th1 cells were collected on day 4–6 of recall response. After density centrifugation on Histopaque 1083 (Sigma-Aldrich), chemotaxis assays were performed using 5-µm pore polycarbonate Transwell filters (Corning Costar). 5×10^5^ cells in 100 µl assay medium were added on fibronectin-coated filters (10 µg/ml, Invitrogen), chemokine dilutions (10 nM CCL4, 10 nM CCL19, 20 nM CXCL12, 100 nM CXCL9) or medium were given to lower chambers. For transmigration through endothelium, 5×10^4^ mlEND1 cells (gift from Prof. Dr. Rupert Hallmann, University Hospital, Lund) in medium were seeded on fibronectin-coated filters and incubated for 48 hours before adding Th1 cells. Migrated cells were recovered after 90 min of incubation at 37°C and frequency of migrated cells was determined by staining with anti-CD4 (L3T4, BD Biosciences) and comparing numbers to a fixed amount of fluorescent beads (Fluoresbrite Microparticles 20 µm, Polysciences Inc.) by flow cytometry [52]. All determinations were performed in triplicate. Chemotactic index indicates the ratio of cells migrated to chemokine versus medium. Confluence of the endothelial monolayer was checked by microscopic analysis. To do so, cells were washed in PBS, fixed with paraformaldehyde, stained with GIEMSA-solution for 30 min. and dried over night. Membranes with cells were removed from the inserts and embedded in Entellan.

### Migration of lymphocytes in vivo

*In vitro* cultured Th1 cells from OVA-TCR^tg^ CD152^−/−^ and CD152^+/+^ were collected on day 5 of recall response. Cells were radioactively labeled with 20 µCi/ml sodium (^51^Cr) chromate (Hartmann Analytic) for 1 hour at 37°C. After washing and dead cell removal, 5×10^6^ cells in 200 µl HBSS per mouse were injected i.v. into tail veins in seven C57/B6 mice per group. 24 hours later, delayed type hypersensitivity (DTH) response was initiated by administration of OVA-peptide (250 ng) in IFA (Pierce) s.c. into one footpad. Injection of PBS/IFA in the other footpad served as control. 48 hours after cell transfer mice were sacrificed and organs were analyzed by Beckmann γ-Counter for recovered radioactivity. Radioactivity in different organs was calculated as percentage of total amount of radioactivity per mouse.

### Statistical Analysis

Statistical analysis was performed using the two-tailed unpaired Student's t-Test (Figure 1–5) or Mann-Whitney Test (Figure 7A, B). p<0.05 (*), p<0.01 (**), p<0.001 (***) were considered significantly different.

## References

[1] Campbell DJ, Kim CH, Butcher EC (2003). Chemokines in the systemic organization of immunity.. Immunological Reviews.

[2] Hamann A, Engelhardt B (2005). Leukocyte trafficking.

[3] Szabo SJ, Sullivan BM, Peng SL, Glimcher LH (2003). Molecular mechanisms regulating Th1 immune responses.. Annual Review of Immunology.

[4] Olsson T (1995). Critical Influences of the Cytokine Orchestration on the Outcome of Myelin Antigen-Specific T-Cell Autoimmunity in Experimental Autoimmune Encephalomyelitis and Multiple-Sclerosis.. Immunological Reviews.

[5] Casares D, Brumeanu TD (2001). Insights into the pathogeneses of type I diabetes: a hint for novel immunospecific therapies.. Curr Mol Med.

[6] Simon AK, Seipelt E, Sieper J (1994). Divergent T-Cell Cytokine Patterns in Inflammatory Arthritis.. Proceedings of the National Academy of Sciences of the United States of America.

[7] Brunner M, Larsen S, Sette A, Mitchison A (1995). Altered Th1/Th2 balance associated with the immunosuppressive/protective effect of the H-2A(b) allele on the response to allo-4-hydroxyphenylpyruvate dioxygenase.. European Journal of Immunology.

[8] Powrie F, Coffman RL (1993). Cytokine Regulation of T-Cell Function - Potential for Therapeutic Intervention.. Immunology Today.

[9] Brunner MC, Chambers CA, Chan FK, Hanke J, Winoto A (1999). CTLA-4-Mediated inhibition of early events of T cell proliferation.. J Immunol.

[10] Kirchhoff S, Muller WW, Li-Weber M, Krammer PH (2000). Up-regulation of c-FLIPshort and reduction of activation-induced cell death in CD28-co-stimulated human T cells.. European Journal of Immunology.

[11] Pandiyan P, Gartner D, Soezeri O, Radbruch A, Schulze-Osthoff K (2004). CD152 (CTLA-4) determines the unequal resistance of Th1 and Th2 cells against activation-induced cell death by a mechanism requiring PI3 kinase function.. J Exp Med.

[12] Kolar P, Knieke K, Hegel JK, Quandt D, Burmester GR (2009). CTLA-4 (CD152) controls homeostasis and suppressive capacity of regulatory T cells in mice.. Arthritis Rheum.

[13] Hoff H, Knieke K, Cabail Z, Hirseland H, Vratsanos G (2009). Surface CD152 (CTLA-4) Expression and Signaling Dictates Longevity of CD28null T Cells.. JI.

[14] Chambers CA, Krummel MF, Boitel B, Hurwitz A, Sullivan TJ (1996). The role of CTLA-4 in the regulation and initiation of T-cell responses.. Immunol Rev.

[15] Egen JG, Kuhns MS, Allison JP (2002). CTLA-4: new insights into its biological function and use in tumor immunotherapy.. Nat Immunol.

[16] Maszyna F, Hoff H, Kunkel D, Radbruch A, Brunner-Weinzierl MC (2003). Diversity of clonal T cell proliferation is mediated by differential expression of CD152 (CTLA-4) on the cell surface of activated individual T lymphocytes.. J Immunol.

[17] Linsley PS, Bradshaw J, Greene J, Peach R, Bennett KL (1996). Intracellular trafficking of CTLA-4 and focal localization towards sites of TCR engagement.. Immunity.

[18] Egen JG, Allison JP (2002). Cytotoxic T lymphocyte antigen-4 accumulation in the immunological synapse is regulated by TCR signal strength.. Immunity.

[19] Gimsa U, Oren A, Pandiyan P, Teichmann D, Bechmann I (2004). Astrocytes protect the CNS: antigen-specific T helper cell responses are inhibited by astrocyte-induced upregulation of CTLA-4 (CD152).. Journal of Molecular Medicine-Jmm.

[20] Dudda JC, Lembo A, Bachtanian E, Huehn J, Siewert C (2005). Dendritic cells govern induction and reprogramming of polarized tissue-selective homing receptor patterns of T cells: important roles for soluble factors and tissue microenvironments.. European Journal of Immunology.

[21] Mora JR, Cheng GY, Picarella D, Briskin M, Buchanan N (2005). Reciprocal and dynamic control of CD8 T cell homing by dendritic cells from skin- and gut-associated lymphoid tissues.. Journal of Experimental Medicine.

[22] Murdoch C (2000). CXCR4: chemokine receptor extraordinaire.. Immunol Rev.

[23] Mackay CR (2001). Chemokines: immunology's high impact factors.. Nat Immunol.

[24] Stein JV, Rot A, Luo Y, Narasimhaswamy M, Nakano H (2000). The CC chemokine thymus-derived chemotactic agent 4 (TCA-4, secondary lymphoid tissue chemokine, 6Ckine, exodus-2) triggers lymphocyte function-associated antigen 1-mediated arrest of rolling T lymphocytes in peripheral lymph node high endothelial venules.. J Exp Med.

[25] Okada T, Ngo VN, Ekland EH, Forster R, Lipp M (2002). Chemokine requirements for B cell entry to lymph nodes and Peyer's patches.. J Exp Med.

[26] Warnock RA, Campbell JJ, Dorf ME, Matsuzawa A, McEvoy LM (2000). The role of chemokines in the microenvironmental control of T versus B cell arrest in Peyer's patch high endothelial venules.. J Exp Med.

[27] Chambers CA, Sullivan TJ, Truong T, Allison JP (1998). Secondary but not primary T cell responses are enhanced in CTLA-4-deficient CD8+ T cells.. Eur J Immunol.

[28] Schenkel AR, Mamdouh Z, Muller WA (2004). Locomotion of monocytes on endothelium is a critical step during extravasation.. Nature Immunology.

[29] Murphy PM (1994). The Molecular-Biology of Leukocyte Chemoattractant Receptors.. Annual Review of Immunology.

[30] Krummel MF, Allison JP (1995). CD28 and CTLA-4 have opposing effects on the response of T cells to stimulation.. J Exp Med.

[31] Walunas TL, Lenschow DJ, Bakker CY, Linsley PS, Freeman GJ (1994). CTLA-4 can function as a negative regulator of T cell activation.. Immunity.

[32] Krummel MF, Allison JP (1996). CTLA-4 engagement inhibits IL-2 accumulation and cell cycle progression upon activation of resting T cells.. J Exp Med.

[33] Scheffold A, Assenmacher M, Reiners-Schramm L, Lauster R, Radbruch A (2000). High-sensitivity immunofluorescence for detection of the pro- and anti-inflammatory cytokines gamma interferon and interleukin-10 on the surface of cytokine-secreting cells.. Nat Med.

[34] Walunas TL, Bakker CY, Bluestone JA (1996). CTLA-4 ligation blocks CD28-dependent T cell activation.. J Exp Med.

[35] Waterhouse P, Penninger JM, Timms E, Wakeham A, Shahinian A (1995). Lymphoproliferative Disorders with Early Lethality in Mice Deficient in Ctla-4.. Science.

[36] Doyle AM, Mullen AC, Villarino AV, Hutchins AS, High FA (2001). Induction of cytotoxic T lymphocyte antigen 4 (CTLA-4) restricts clonal expansion of helper T cells.. Journal of Experimental Medicine.

[37] Scheffold A, Miltenyi S, Radbruch A (1995). Magnetofluorescent liposomes for increased sensitivity of immunofluorescence.. Immunotechnology.

[38] Brunner-Weinzierl MC, Hoff H, Burmester GR (2004). Multiple functions for CD28 and cytotoxic T lymphocyte antigen-4 during different phases of T cell responses: implications for arthritis and autoimmune diseases.. Arthritis Research & Therapy.

[39] Schneider H, Mandelbrot DA, Greenwald RJ, Ng F, Lechler R (2002). Cutting edge: CTLA-4 (CD152) differentially regulates mitogen-activated protein kinases (extracellular signal-regulated kinase and c-Jun N-terminal kinase) in CD4(+) T cells from receptor/ligand-deficient mice.. Journal of Immunology.

[40] Schneider H, Valk E, Dias SD, Wei B, Rudd CE (2005). CTLA-4 up-regulation of lymphocyte function-associated antigen 1 adhesion and clustering as an alternate basis for coreceptor function.. Proceedings of the National Academy of Sciences of the United States of America.

[41] Schneider H, Downey J, Smith A, Zinselmeyer BH, Rush C (2006). Reversal of the TCR stop signal by CTLA-4.. Science.

[42] Mustelin T (2006). Restless T cells sniff and go.. Science.

[43] Bleul CC, Schultze JL, Springer TA (1998). B Lymphocyte Chemotaxis Regulated in Association with Microanatomic Localization, Differentiation State, and B Cell Receptor Engagement.. J Exp Med.

[44] Fedyk ER, Ryyan DH, Ritterman I, Springer TA (1999). Maturation decreases responsiveness of human bone marrow B lineage cells to stromal-derived factor 1 (SDF-1).. J Leukoc Biol.

[45] Shen H, Cheng T, Olszak I, Garcia-Zepeda E, Lu Z (2001). CXCR-4 Desensitization Is Associated with Tissue Localization of Hemopoietic Progenitor Cells.. J Immunol.

[46] Thelen M, Stein JV (2008). How chemokines invite leukocytes to dance.. Nat Immunol.

[47] Marengere LE, Waterhouse P, Duncan GS, Mittrucker HW, Feng GS (1996). Regulation of T cell receptor signaling by tyrosine phosphatase SYP association with CTLA-4.. Science.

[48] Debes GF, Arnold CN, Young AJ, Krautwald S, Lipp M (2005). Chemokine receptor CCR7 required for T lymphocyte exit from peripheral tissues.. Nature Immunology.

[49] Bromley SK, Thomas SY, Luster AD (2005). Chemokine receptor CCR7 guides T cell exit from peripheral tissues and entry into afferent lymphatics.. Nature Immunology.

[50] Gett AV, Sallusto F, Lanzavecchia A, Geginat J (2003). T cell fitness determined by signal strength.. Nature Immunology.

[51] Foxman EF, Kunkel EJ, Butcher EC (1999). Integrating conflicting chemotactic signals: The role of memory in leukocyte navigation.. Journal of Cell Biology.

[52] Debes GF, Hopken UE, Hamann A (2002). In vivo differentiated cytokine-producing CD4(+) T cells express functional CCR7.. Journal of Immunology.

[53] Reape TJ, Rayner K, Manning CD, Gee AN, Barnette MS (1999). Expression and cellular localization of the CC chemokines PARC and ELC in human atherosclerotic plaques.. American Journal of Pathology.

[54] Hjelmstrom P, Fjell J, Nakagawa T, Sacca R, Cuff CA (2000). Lymphoid tissue homing chemokines are expressed in chronic inflammation.. American Journal of Pathology.

[55] Weninger W, Carlsen HS, Goodarzi M, Moazed F, Crowley MA (2003). Naive T cell recruitment to nonlymphoid tissues: A role for endothelium-expressed CC chemokine ligand 21 in autoimmune disease and lymphoid neogenesis.. Journal of Immunology.

